# Microstructural Characterization, Tribological and Corrosion Behavior of H111 Hot-Rolled AA5754 after Homogenization and Aging

**DOI:** 10.3390/ma17133164

**Published:** 2024-06-27

**Authors:** Otman Farj Mohammed Abukhdair, Ismail Esen, Hayrettin Ahlatci, Esma Keskin

**Affiliations:** 1 Mechanical Engineering Department, Karabuk University, Karabuk 78050, Turkey; 2138169048@ogrenci.karabuk.edu.tr (O.F.M.A.); iesen@karabuk.edu.tr (I.E.); 2 Metallurgical and Materials Engineering Department, Karabuk University, Karabuk 78050, Turkey

**Keywords:** AA5754, wear resistance, corrosion behavior, microstructure

## Abstract

In this study, the microstructural properties, wear resistance, and corrosion behavior of H111 hot-rolled AA5754 alloy before heat treatment, after homogenization, and after aging were examined. The microstructure was mainly composed of the scattered forms of black and gray contrast particles on the matrix and precipitations were observed at the boundaries of the grain. The as-rolled material exhibited a dense pancake-shaped grain structure, which is typical of as-rolled material. Observation along the L-direction did not yield distinct demarcations among the grains and was not uniformly distributed, with precipitates at the grain boundary. When they aged, there was a parallel increase in fine and huge black and gray contrast particles in the zone. Therefore, it could be stated that the amount of fine grains increased due to the rise in the homogenization process. The rolled base metal with the grain orientation was found to be parallel to the rolling direction. On the other hand, the coarse grains were clearly observed in the aging heat-treatment condition. The grains had an elongated morphology consistent with the rolling process of the metal before the heat-treatment process. The aged alloy had the highest hardness with a value of 86.83 HB; the lowest hardness was seen in the alloy before heat treatment with a value of 68.67 HB. The weight loss and wear rate of this material at the end of 10,000 m were, respectively, 1.01 × 10^−3^ g and 5.07 × 10^−9^ g/Nm. It was observed that the alloy had the highest weight loss and worst wear resistance before heat treatment. Weight loss and wear rates at the end of 10,000 m were, respectively, 3.42 × 10^−3^ g and 17.08 × 10^−9^ g/Nm. According to these results, the friction coefficients during wear were parallel and the material with the lowest friction coefficient after aging was 0.045. While the alloys corroded after aging showed more weight loss, the alloys corroded before heat treatment exhibited better corrosion behavior. Among the alloys, the least weight loss after 24 h was observed in the alloy that was corroded before heat treatment and this value was 0.69 × 10^−3^ mg/dm^2^. The highest weight loss was observed in the aged alloy with a value of 1.37 × 10^−3^ mg/dm^2^. The alloy before heat treatment, which corroded after casting, showed the lowest corrosion rate with a value of 0.39 × 10^−3^ mg/(dm^2^·day) after 72 h. The alloy that was corroded before heat treatment showed the best corrosion behavior by creating a corrosion potential of 1.04 ± 1.5 V at a current density of −586 ± 0.04 μA/cm^2^. However, after aging, the corroded alloy showed the worst corrosion behavior with a corrosion potential of 5.16 ± 3.3 V at a current density of −880 ± 0.01 μA/cm^2^.

## 1. Introduction

The development of the non-ferrous metal alloys industry will continue to expand as the demand for more technologically complex and ecologically sustainable materials increases. In recent years, the use of light and durable materials has been increasing in many areas [1]. As one of the youngest members of the global non-ferrous metals, aluminum’s unique properties—its light weight, high strength, and resistance to corrosion—make it an ideal material for use in traditional and new applications [2]. Aluminum alloys, especially AA5754 Al-Mg alloys, are widely used due to their high ductility and durability [3], good weldability, good corrosion resistance, and low density [4]. AA5754 is an Mg-rich aluminum alloy commonly used in ship hauling and tread plate flooring applications. Additionally, the wear behavior of AA5754 alloy is of interest in various industrial applications where components are exposed to abrasive or sliding wear conditions. Therefore, the friction wear criteria is an important area of study for this alloy [5]. However, regardless of its good corrosion resistance, it is often severely damaged under the synergistic attack of wear and corrosion in some aggressive environments [6]. Since poor tribological performance limits the use of aluminum and its alloys in wear-related applications, mass modification [7,8] and the improvement of surface properties [9,10,11,12] have been achieved to improve wear and corrosion resistance. When considering the wear of the AA5754 alloy, it is essential to understand the mechanisms that govern wear in this material. Wear in metals like AA5754 can occur through various mechanisms, including adhesive wear and abrasive wear. Adhesive wear happens when two surfaces slide against each other, causing material transfer between the surfaces. Abrasive wear occurs due to the presence of hard particles or asperities that plough into the softer material, causing material removal. The wear resistance of AA5754 alloy can be influenced by several factors, including the microstructure of the material, surface treatments, environmental conditions, and the type of wear mechanism predominant in the application. The microstructure of AA5754, which includes the distribution of second-phase particles and grain size, plays a significant role in determining its wear resistance [13]. In Al–Mg alloys, magnesium (supersaturated) with 5% Mg content by weight (e.g., 5086, 5754, and 5456, etc.) causes strong solution hardening effects [14]. When exposed to high temperatures for a long time, it precipitates at dislocations and grain boundaries (mainly β-phase, Al_3_Mg_2_, fcc [15]) [16]. Thus, any mechanism that prevents the movements of dislocations increases the hardness, yield strength, and tensile strength of the alloy. The high strength of an age-hardened alloy is due to the interaction of the precipitated second-phase particles with dislocations. As a result, coherent second-phase precipitation constitutes the mechanism providing strength increase [17]. The hardening mechanism of this type of Al-Mg alloy is due to the formation of the β phase [18]. Excess Mg atoms in the matrix are supersaturated atoms and the remaining Mg atoms are in the β-phase form [19,20,21,22,23,24]. The type, distribution, amount, average diameter, and number of precipitated secondary phases affect the strength value [17]. The smaller, denser, and more homogeneous the precipitated secondary phase particles, the higher the strength.

Several studies have investigated the wear and corrosion properties of the AA5754 alloy under different conditions. Chen et al. [25] investigated fretting wear in AA5754 joints joined by self-piercing riveting (SPR), emphasizing the impact of this joining method on wear characteristics. Zhao et al. [26] further explored the influence of sheet thickness on fatigue behavior and fretting in self-piercing riveted joints, providing insights into how different parameters affect wear in similar aluminum alloys. Additionally, Ang [27] discussed joint failures and corrosion issues in SPR joints, shedding light on fretting wear locations and common forms of corrosion in AA5754 joints. Moreover, the study by Ruiz-Andrés et al. [28] delved into the wear behavior of aluminum alloys at slow sliding speeds, including AA5754, highlighting the importance of understanding wear mechanisms under different conditions. Furthermore, Luri et al. [29] analyzed fatigue and wear behavior in ultrafine-grained connecting rods made from AA5754, showcasing the significance of material processing techniques on wear properties. Afshar et al. [30] investigated the impact of heat-treatment and cleaning processes on the filiform corrosion resistance of AA5754 alloy. Furthermore, Al-Helal et al. [31] emphasized the excellent corrosion resistance of AA5754 alloy, making it a suitable candidate for recycling due to its properties. Additionally, Ahmed et al. [32] highlighted that AA5754, being an aluminum–magnesium alloy, is known for its high corrosion resistance and good formability.

Overall, the wear behavior of the AA5754 alloy is influenced by various factors including applied load, sliding speed, surface roughness, presence of abrasive particles, and alloying elements. Understanding these factors is crucial for optimizing the wear resistance of the AA5754 alloy in practical applications. Moreover, the corrosion behavior of the AA5754 alloy is influenced by factors such as microstructure, the presence of certain elements like iron, and the size of precipitates. These characteristics contribute to the alloy’s resistance to various forms of corrosion, making it a preferred choice for applications where corrosion resistance is crucial. Further research is needed to explore advanced surface treatments and alloy modifications that can further enhance the wear and corrosion properties of this alloy.

Overall, the wear behavior of the AA5754 alloy is influenced by various factors including applied load, sliding speed, surface roughness, presence of abrasive particles, and alloying elements. Understanding these factors is crucial for optimizing the wear resistance of the AA5754 alloy in practical applications. Moreover, the corrosion behavior of the AA5754 alloy is influenced by factors such as microstructure, the presence of certain elements like iron, and the size of precipitates. These characteristics contribute to the alloy’s resistance to various forms of corrosion, making it a preferred choice for applications where corrosion resistance is crucial. Further research is needed to explore advanced surface treatments and alloy modifications that can further enhance the wear and corrosion properties of this alloy. This research aims to examine the effects of H111 hot-rolled AA5754 alloy on microstructure, corrosion, and wear behavior by comparing the conditions before and after heat treatment (homogenization and aging) and to shed light on the literature.

## 2. Materials and Methods

H111 hot-rolled AA5754 material was supplied from Seykoç Aluminum. The chemical compositions of this material are given in Table 1. The alloys’ X-ray fluorescence (measured using an XRF Rigaku ZSX Primus II, Rigaku, Tokyo, Japan) chemical makeup is laid forth in weight-based Table 1. After casting, the phases of the alloy were determined by X-ray diffractometry (XRD Rigaku Ultima IV, Rigaku, Tokyo, Japan) at 10–90° and 3°/min.

Protherm PLF 120/5 brand heat-treatment furnace was used for the homogenization process. The rectangular-shaped materials with the size of 140 × 124 × 10, which were cut in the rolling direction, and were covered with aluminium foil. Sample directions are given as L for the long direction (the rolling direction), ST for the short transverse direction (the thickness of the plate), and LT for the long transverse (the width of the plate). The as-rolled sheets were subjected to one of two heat treatments in this study: (1) homogenization at 500 °C for 24 h; (2) aging at 150 °C for 72 h. All heat treatments were terminated by quenching the samples into water. The homogenization process was carried out in SiO_2_ + Graphite sand at 500 °C for 24 h and the homogenized alloys were removed from the furnace and cooled in water. After homogenization, the aging process was applied to half of the rectangular-shaped materials (70 × 62 × 10 in size) with the size of 140 × 124 × 10. The aging process was carried out in the same heat-treatment furnace at 150 °C for 72 h. Samples of 10 × 10 × 10 mm were prepared to determine the microstructural characterization before heat treatment, after homogenization, and after aging. Wet sanding was performed by using 400–2500 mesh silicon carbide-containing sandpaper in Mikrotest brand sanding and polishing device. At the end of the sanding, 3 μm alumina paste was used for polishing. Keller etching (2 mL HF, 3 mL HCl, 5 mL HNO_3_ and 190 mL pure H_2_O) has characterized the microstructure of the alloys before heat treatment, after homogenization, and after aging. The optical microscope was used to see the change in the grains in the phase structure, while SEM and EDX have performed to reveal the secondary phases. Brinell hardness was measured at 750 N with 5 mm steel balls.

Tribological testing was performed at room temperature with the loading axis parallel to the rolling direction (L). For wear tests, rectangular prism-shaped samples with sides of 18 mm and 14 mm in length and 10 mm in height were prepared. The surfaces intended for wear were sanded using 1200 μm sandpaper. The weights of the samples, which were cleaned with ethanol, were measured using a Precisa brand balance with an accuracy of 0.1 mg. Abrasion tests were conducted using a reciprocating wear test apparatus in a dry setting, with a 20 N force, a sliding speed of 0.1 m/s, and a total sliding distance of 10,000 m. An AISI 52100 high-hardness steel ball is utilized as the abrasive tip material. Every 200 m, ethanol was utilized to cleanse the sample surface of wear residues. The samples cleaned with ethanol were weighed using precision scales and subjected to the back-and-forth abrasion test once again. The weight reductions based on the distance were determined by subtracting the pre-wear value from the post-wear value using these data. The mass loss data was transformed into a particular wear rate using Equation (1).

(1) $$\mathrm{Specific}\;\mathrm{Wear}\;\mathrm{Rate}\left(g/N\cdot m\right)=\frac{\mathrm{Wear}\;\mathrm{Mass}\;\mathrm{Loss}\;\left(g\right)}{\mathrm{Applied}\;\mathrm{Load}\;\left(N\right)\times \mathrm{Sliding}\;\mathrm{Distance}\;\left(m\right)}.\;$$

The friction force during wear was measured using a load cell linked to the tribometer arm and promptly recorded on the computer. The wear mechanism was studied using SEM and EDX instruments to analyse changes in alloy element concentration and wear load throughout the wear test.

Square prism-shaped samples with an approximate edge length of 12 mm and a height of 10 mm were used in the immersion corrosion test. Surface area and weight measurements were undertaken for each sample using a Precisa precision balance. Immersion corrosion lasted 12–72 h in 3.5% NaCl. Chromic acid and ethanol, produced from 180 g of CrO_3_ and 1 L of distilled water, were utilized to remove corrosion residues from the sample surface every hour. The corrosion samples were initially immersed in chromic acid for approximately 3 min, followed by rinsing with pure water to remove the acid residue. The corrosion samples were rinsed with distilled water, immersed in ethanol for approximately 2 min, and then dried. Following the drying process, the corrosion samples were weighed individually using precision scales and then subjected to immersion corrosion once again. The weight losses in grams observed at each hour interval were translated into milligrams due to the immersion corrosion process. The corrosion sample’s surface area in mm^2^ was converted to dm^2^. The milligram lost per square decimeter per hour was calculated by dividing the average weight loss (mg) by the surface area (dm^2^). Weight losses were calculated by subtracting the initial value from the final amount. The weight loss after 24 h was calculated by subtracting the initial weight/surface area value from the weight/surface area value recorded after 24 h.The corrosion rate was determined in milligrams per square decimeter per day (mg/dm^2^·day). Corrosion rates per day were calculated by dividing the weight losses by the number of days. The weight loss after 72 h was divided by 3 to calculate the corrosion rate corresponding to 3 days. The computerized DC105 Gamry type PC4/300 mA potentiostat/galvanostat device conducted the potentiodynamic polarization test. The experiment used a 3.5% sodium chloride solution. By adjusting the corrosion potential, scanning at ±0.25 mV at a rate of 1 mVs^−1^ produced tafel curves. Potentiodynamic polarization corrosion tests are square prism-shaped samples wrapped with copper wire and cold mounted (as working electrode). The samples were sanded using 2500 µm sandpaper for testing, and tapes having a surface area of 0.19 cm^2^ were then affixed to the sample surfaces. The saturated calomel electrode (SCE) and graphite rod were used as reference and counter electrodes, respectively. The samples underwent potentiodynamic polarization corrosion testing. Two duplicate samples were utilized to assess corrosion characteristics in immersion corrosion and potentiodynamic polarization corrosion experiments. Twelve samples were utilized for corrosion testing.

## 3. Results and Discussion

### 3.1. XRD Patterns

**Figure 1:**
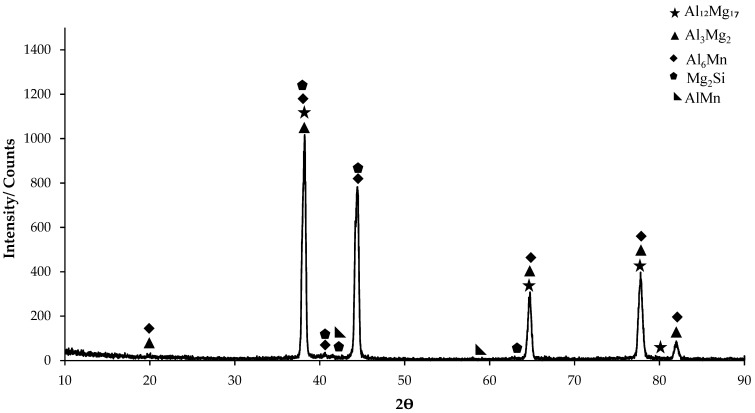
X-ray diffraction (XRD) pattern of H111 hot-rolled AA5754 alloy.

In the XRD standard card (Figure 1), predominantly Al_6_Mn phase is seen. AlMn phase is also seen as small peaks on the XRD card. On the XRD card of H111 hot-rolled AA5754 alloy, the first phases at 20° were determined as Al_3_Mg_2_ and Al_6_Mn. The highest peak occurred at 38° and the peaks here were Al_3_Mg_2_, Al_12_Mg_17_, Mg_2_Si, and Al_6_Mn phases. While it ended at 82° (Figure 1), the phases occurring at 20° were seen again on the XRD standard card.

### 3.2. Microstructure

**Figure 2:**
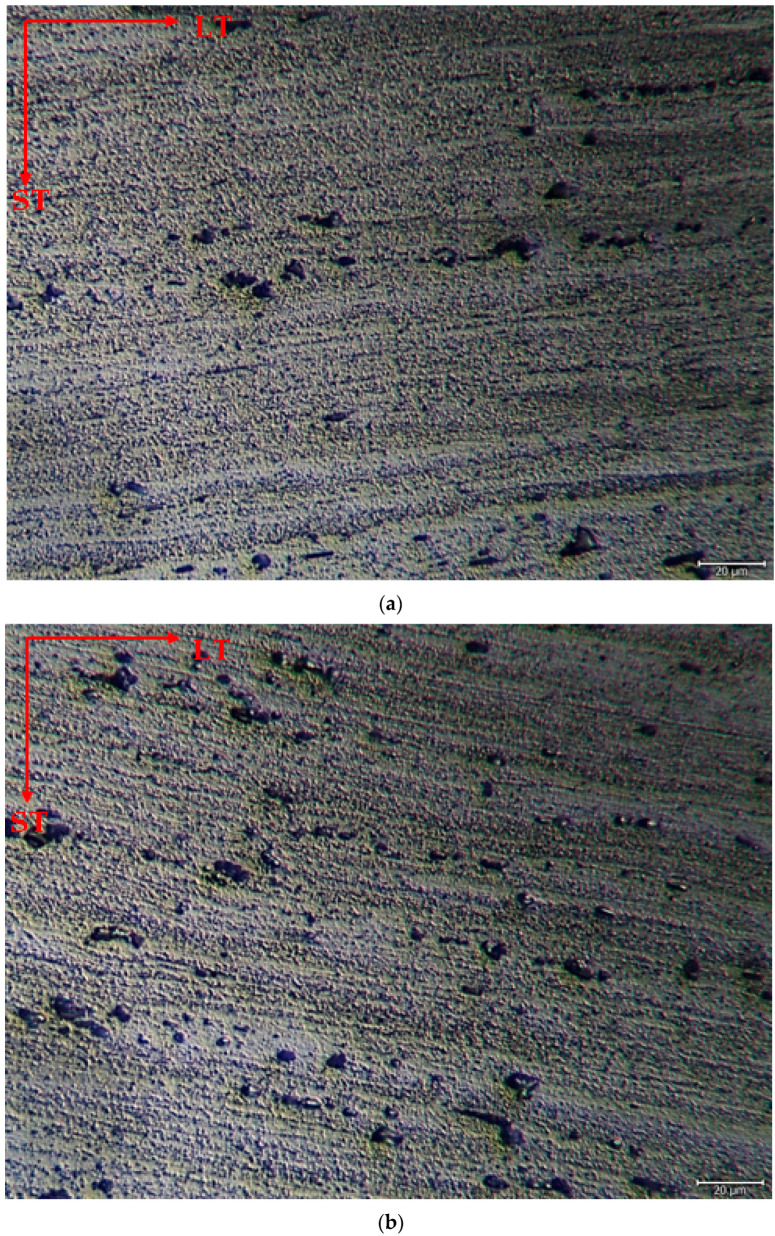

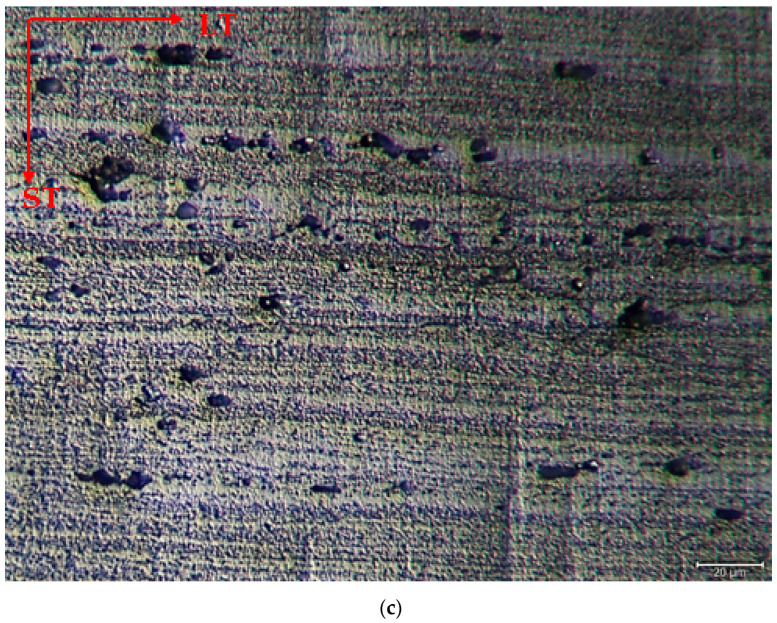
Image of H111 hot-rolled AA5754 alloy LOM: (**a**) No heat treatment, (**b**) Homogenized, and (**c**) Aged.

The microstructure was mainly composed of the scattered forms of black and gray contrast particles on the matrix and precipitations were observed at the boundaries of the grain. The as-rolled material exhibited a dense pancake-shaped grain structure, which is typical of as-rolled material (Figure 2). Observation along the L-direction did not yield distinct demarcations among grains and was not uniformly distributed, with precipitates at the grain boundary. When they aged, there was a parallel increase in fine and huge black and gray contrast particles in the zone. Therefore, it could be stated that the amount of fine grains increased due to the rise in the homogenization process. (Figure 2b). The rolled base metal with the grain orientation was found to be parallel to the rolling direction. On the other hand, the coarse grains were clearly observed in the aging heat-treatment condition (Figure 2c). The grains have an elongated morphology consistent with the rolling process of the metal before the heat-treatment process. The aging process can lead to changes in the microstructure, tribological, and corrosive properties of these alloys, affecting their resistance. Research has shown that the unconventional behavior of 5XXX alloys at different temperatures and strain rates can be attributed to dynamic strain aging effects [33].

**Figure 3:**
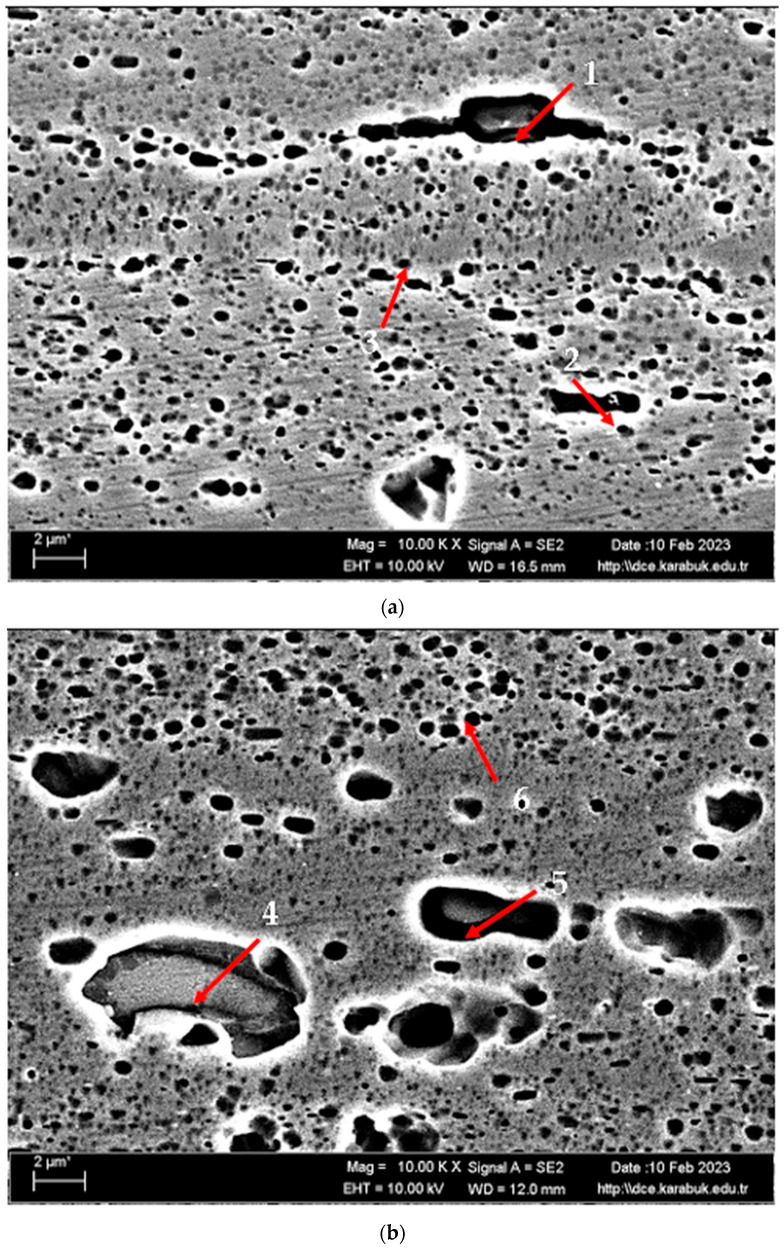

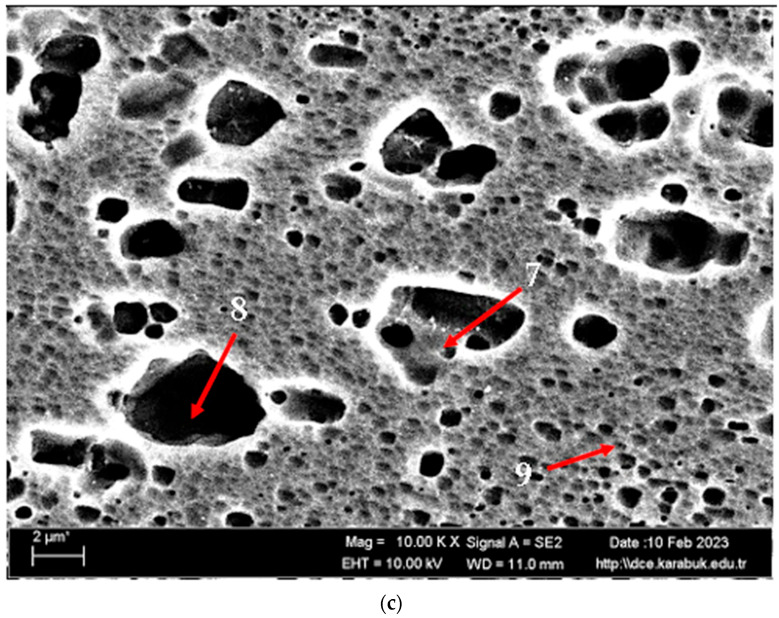
Image of H111 hot-rolled AA5754 alloy SEM: (**a**) No heat treatment, (**b**) Homogenized, and (**c**) Aged.

Figure 3 shows the SEM micrographs of the H111 hot-rolled AA5754 alloy before heat treatment, after homogenization, and after aging. Table 2 shows the EDX analysis of the second stage with different morphologies labelled (1–9) in Figure 3a–c. In general, it is possible to say that the presence of silicon increases with the effect of heat treatment. It is thought that the gray-contrast-colored cylindrical structure at point 1 in Figure 3a and the small white structures in this structure are composed of aluminium-rich Al_12_Mg_17_ and AlMn phase. While the presence of silicon and manganese number 2 is not observed, the bright white small triangular structure formed here is believed to be the Al_12_Mg_17_ phase (see EDX (Table 2)). The presence of manganese was not seen at point 3 either. However, while the small Al_12_Mg_17_ phase spread in the matrix continued to exist, a small amount of Mg_2_Si was formed, which was also seen in the EDX analysis. Point 4 in Figure 3b is at the point where manganese presence is at its peak, as seen in the EDX analysis. Here, the capillary-shaped structures with gray contrast and white color on the matrix surface are likely to be Al_6_Mn, AlMn, Al_3_Mg_2_, and Mg_2_Si. At point 5, there is a similar situation to point 3. It is likely that there are small white/gray contrast Al_12_Mg_17_ and Mg_2_Si phases here as well. While there is no silicon presence at point 6, AlMn and Al_12_Mg_17_ phases are distributed around the dark-gray contrasting ellipse-shaped structures. In Figure 3c, point 7 is the richest place in the silicon. It is possible that the small cube-shaped white structures at the grain and grain boundaries are Al_3_Mg_2_, AlMn, and Mg_2_Si intermetallics. While very little silicon and manganese are found at point 8, it is thought that mostly Al_12_Mg_17_ phase is formed here. At point 9, the density of capillary-shaped structures with gray contrast and white color on the matrix surface has increased and become denser. These structures are thought to be Al_12_Mg_17_, AlMn, and Mg_2_Si phases.

### 3.3. Hardness Test Results

**Figure 4:**
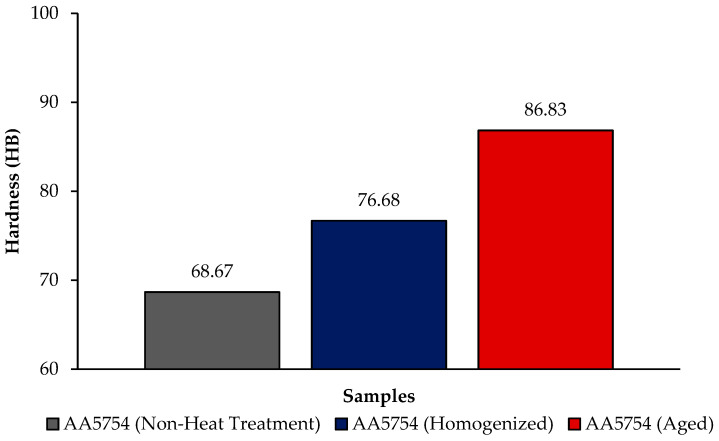
Hardness results of H111 hot-rolled AA5754 alloy of before heat treatment, after homogenization and aging.

Figure 4 shows the comparison of H111 hot-rolled AA5754 alloy in terms of hardness before heat treatment, after homogenization, and after aging. In general, there is an increase in hardness data after heat treatment. Homogenization plays a critical role in determining the hardness of aluminum alloys. This is believed to be due to evenly distributed secondary stages. Aging significantly influences the hardness of aluminum alloys. Pre-aging has been demonstrated to amplify the impact of artificial aging on the hardness and strength of aluminum alloys [34]. The formation of precipitates during aging, such as Mg_2_Si, Al_2_Cu, and AlFeSi, substantially contributes to the enhancement of hardness and wear resistance in 5XXX series aluminum alloys [35]. As can be seen from Figure 4, the aged alloy has the highest hardness with a value of 86.83 HB; the lowest hardness was seen in the alloy before heat treatment with a value of 68.67 HB.

A range of studies have investigated the microstructural changes and mechanical properties of various aluminum alloys during heat treatment. Fuller [36] found that the addition of Sc and Zr to a 5754 aluminum alloy led to the formation of two types of precipitates, which had contrasting effects on the alloy’s mechanical properties. Similarly, Ghosh [37] observed that the addition of Ag and Sn to a 7075 aluminum alloy resulted in the formation of fine precipitates, which improved the alloy’s strength and ductility. Hussain et al. [38] and Han et al. [39] both studied the effects of processing and aging on the microstructure and mechanical properties of aluminum alloys, finding that these factors can significantly influence the size, density, and distribution of precipitates, and consequently, the alloy’s mechanical properties. The initial strength of the non-heat-treated H111 hot-rolled AA5754 alloy is achieved through a combination of solid solution hardening due to dissolved Mg and dispersion hardening by the dispersoid-forming Mn and Si alloying elements. Thus, the proper control of final material properties requires knowledge of the effect of alloy composition and process parameters on the microstructure of the resulting material and the state of second-phase particles and alloying elements in a solid solution. With the homogenization application to the AA5754 alloy, strengthening occurred due to the development of second-phase particles and the effect of these alloys on static recrystallization. According to the XRD results given in Figure 1, the second-phase particles contributing to strength are thought to be Mg_2_Si, Al_6_(Mn,Fe), and Al(Mn,Fe,Si). These findings were reported by Engler et al. [40,41,42] and are compatible with the studies conducted. The increase in strength of the aging heat-treated H111 hot-rolled AA5754 alloy can be attributed to the homogeneous distribution of Mg_2_Si precipitates within the structure.

### 3.4. Wear Test Results

**Figure 5:**
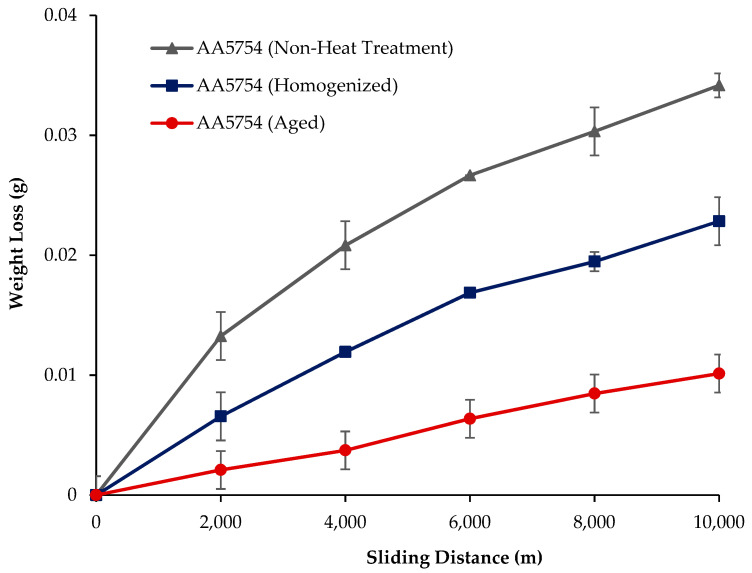
Results of back-and-forth wear weight loss of H111 hot-rolled AA5754 alloy before heat treatment, and after homogenization and aging.

**Figure 6:**
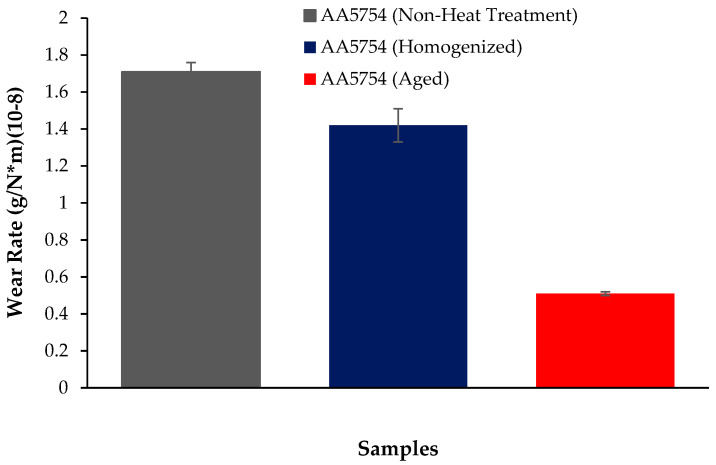
Results of back-and forth-wear rates of H111 hot-rolled AA5754 alloy of before heat treatment, and after homogenization and aging.

**Figure 7:**
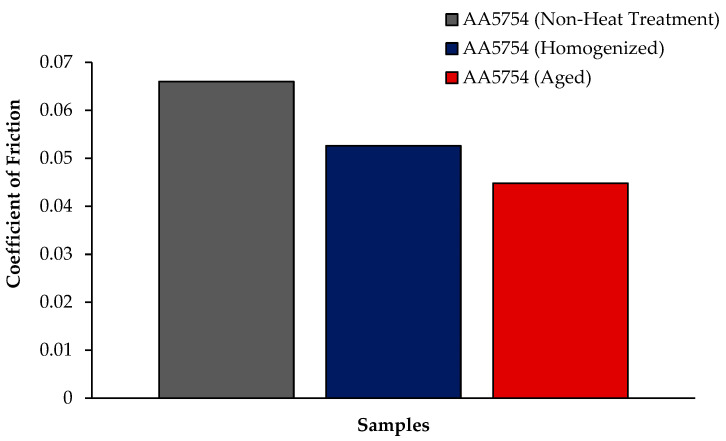
Results of friction coefficient of H111 hot-rolled AA5754 alloy of before heat treatment, and after homogenization and aging.

The variation in the weights of the H111 hot-rolled AA5754 alloy before heat treatment, after homogenization, and after aging according to distance is shown in Figure 5; the wear rates at the end of 10,000 m are given comparatively in Figure 6; and the friction coefficients taken during wear are given in Figure 7. While alloys exposed to corrosion before heat treatment show more weight loss, alloys exposed to wear after aging exhibited better wear resistance. The wear results support the hardness results and there is parallelism in the results. In addition, sensitization caused by precipitations of Mg_2_Si and β-phase Mg_2_Al_3_ in the alloy can have a significant impact on wear resistance. Studies have shown that the β-phase can provide protection during the wear process by resisting plowing actions on the matrix, reducing abrasive wear effects, and enhancing overall wear resistance [43]. Additionally, the dissolution of the β-Mg_17_Al_12_ phase through solid-solution treatment has been shown to significantly enhance the wear resistance of certain alloys [44]. It was determined that the alloy after aging was the material that provided the least weight loss after 10,000 m and had the best wear resistance. The weight loss and wear rate of this material at the end of 10,000 m are, respectively, 1.01 × 10^−3^ g and 5.07 × 10^−9^ g/Nm. It was observed that the alloy had the highest weight loss and worst wear resistance before heat treatment. Weight loss and wear rates at the end of 10,000 m are, respectively, 3.42 × 10^−3^ g and 17.08 × 10^−9^ g/Nm. According to these results, the friction coefficients during wear are parallel and the material with the lowest friction coefficient after aging is 0.045.

**Figure 8:**
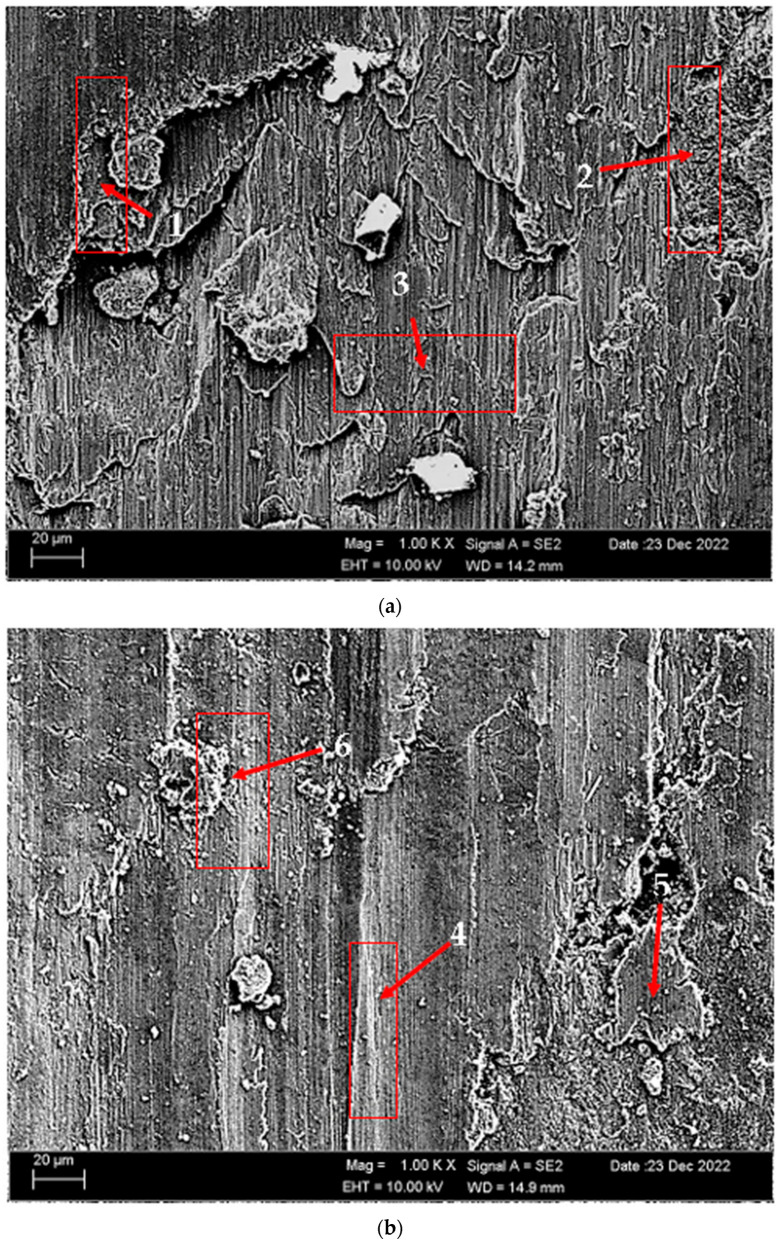

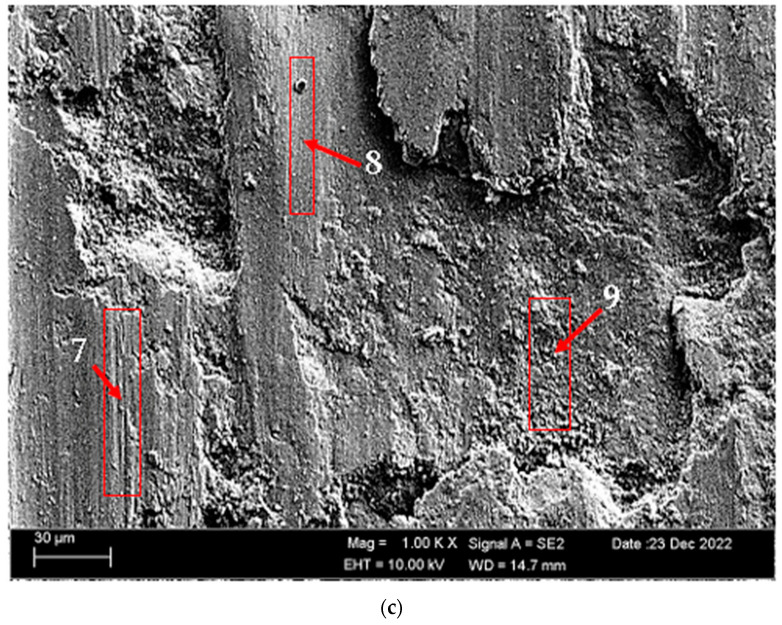
SEM micrographs of wear-tested H111 hot-rolled AA5754 alloys: (**a**) No heat treatment, (**b**) Homogenized, and (**c**) Aged.

According to SEM micrographs, the effective wear mechanism appears to be abrasive and adhesive wear types. According to the wear micrographs obtained, it can be seen that there are deposits caused by plastic deformation around the wear scar, wear grooves are formed, and sharp-line scraping is formed as a result of abrasive wear. Additionally, two-body and three-body wear was observed. In two-body wear, the abrasive particles move across the surface without rotating, while in three-body wear, the abrasive particles rotate freely, leading to different wear mechanisms [45]. In addition, adhesive wear, which involves material transfer between surfaces due to adhesion forces during sliding or contact, is often observed. Figure 8 shows the SEM micrographs of H111 hot-rolled AA5754 alloy before heat treatment, after homogenization and after aging. Table 3 presents the results of the Energy Dispersive X-ray (EDX) study performed in the second stage, and exhibits different morphologies shown as (1–9) in Figure 8a–c. In Figure 8a, it is seen that the deep wear marks at point 1 in the alloy before the heat treatment are broken off in places and the broken pieces stick in the wear scar as debris. At point 2, swollen flakes in the form of sticky crusts are seen on the matrix surface due to the pressure of the abrasive surface. At point 3, the wear marks are deeper and there is a wave appearance caused by broken parts. In Figure 8b, it is seen that particles in the form of small spheres adhere to the wear line at point 4 of the alloy after homogenization. It was determined that the broken pieces at point 5 were stuck within shallow wear lines. At point 6, the broken piece clung to the matrix surface. In Figure 8c, not very deep wear grooves are visible at point 7 of the aged alloy, while a tiny spherical particle is noticeable above this shallow wear line at point 8. At point 9, particles are plastered onto the matrix surface.

### 3.5. Corrosion Behavior

#### 3.5.1. Immersion Test Results

**Figure 9:**
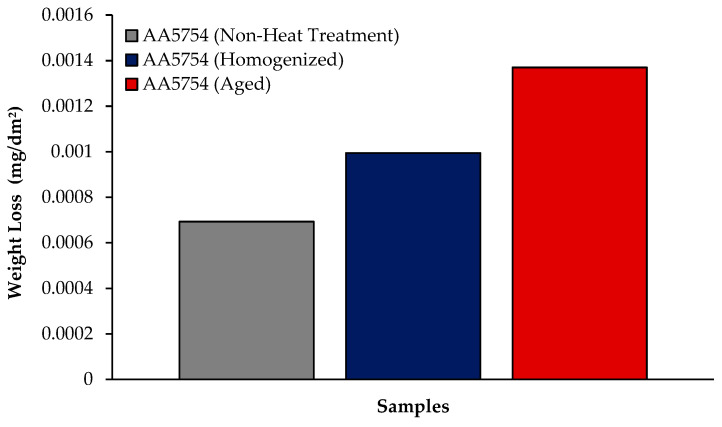
Results of immersion corrosion weight loss of H111 hot-rolled AA5754 alloy before heat treatment, and after homogenization and aging.

**Figure 10:**
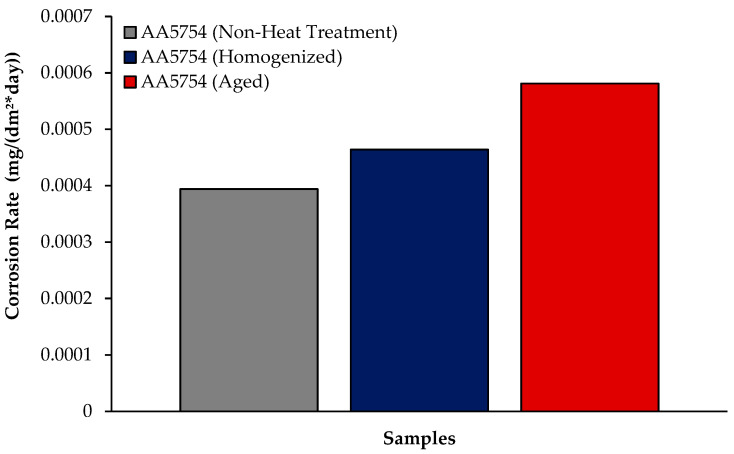
Results of immersion corrosion rates of H111 hot-rolled AA5754 alloy before heat treatment, after homogenization, and after aging.

The changes in 24 h weight loss of H111 hot-rolled AA5754 alloy before heat treatment, and after homogenization and aging is shown in Figure 9; the corrosion rates after 72 h are given comparatively in Figure 10. While the alloys corroded after aging showed more weight loss, the alloys corroded before heat treatment exhibited better corrosion behavior. Among the alloys, the least weight loss after 24 h was observed in the alloy that was corroded before heat treatment and this value was 0.69 × 10^−3^ mg/dm^2^. The highest weight loss was observed in the aged alloy with a value of 1.37 × 10^−3^ mg/dm^2^. The alloy before heat treatment, which corroded after casting, showed the lowest corrosion rate with a value of 0.39 × 10^−3^ mg/(dm^2^·day) after 72 h. Calabrese et al. [46] found that the low sensitivity to pitting corrosion in AA5754 can be attributed to the low content and small size of the precipitates. Additionally, Baqerzadeh Chehreh et al. [47] noted that the presence of a small fraction of iron in AA5754 contributes to its enhanced corrosion resistance in a NaCl solution compared with pure aluminium. However, it is crucial to note that an excessive amount of the segregated β-phase can increase stress corrosion sensitivity [48]. Additionally, sensitization following the precipitations of Mg_2_Si and β-phase Mg_2_Al_3_ can induce pitting, intergranular corrosion, and stress corrosion in 5XXX series aluminium alloys [49]. The presence of sensitization in these alloys has been a subject of study due to its implications for their corrosion resistance [50].

**Figure 11:**
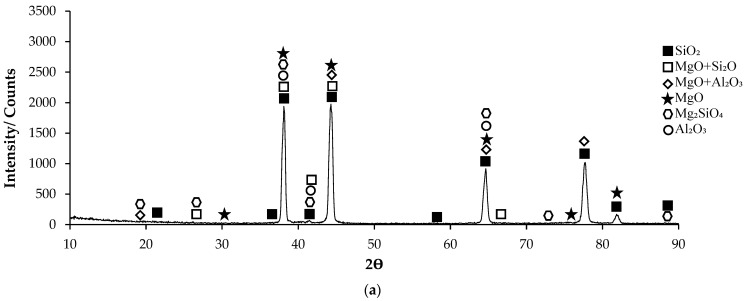

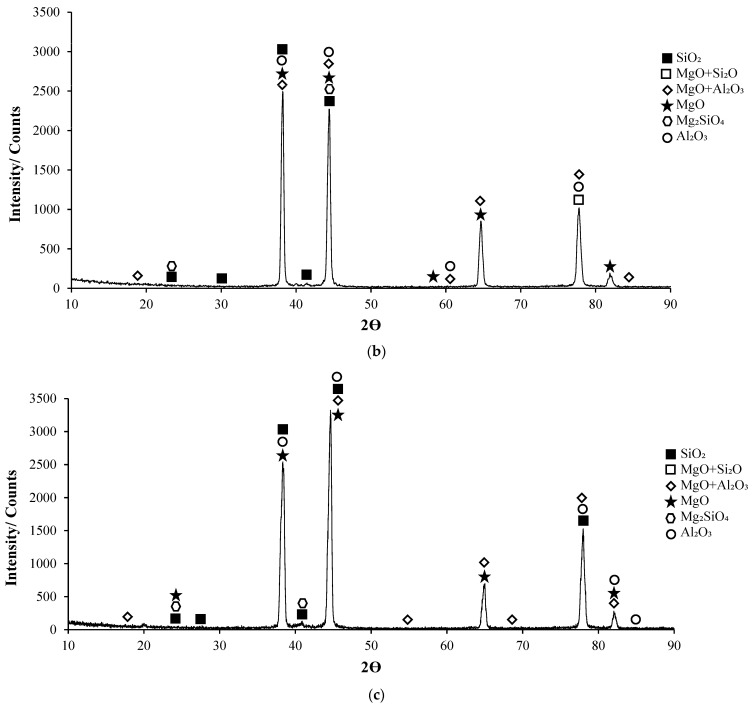
XRD patterns of corroded H111 hot-rolled AA5754 alloys: (**a**) No heat treatment, (**b**) Homogenized, and (**c**) Aged.

Post-corrosion XRD patterns of H111 hot-rolled AA5754 alloy before heat treatment, after homogenization, and after aging are given in Figure 11, respectively. In XRD standard cards, the presence of SiO_2_ in the alloys before heat treatment is noteworthy. When looking at alloys in general, high X-ray diffraction (XRD) intensity was observed in the alloy (3260.83) after aging. X-ray diffraction (XRD) intensity in a diffraction pattern is a crucial parameter that provides information about the crystalline structure of a material. The intensity of peaks in an XRD pattern corresponds to the amount of X-rays diffracted by the crystal planes within the material. When all peak intensities and peak locations match perfectly with a reference card, the XRD score of the analysed mineral is 100, indicating a high degree of similarity with the theoretical compound [51]. Moreover, changes in XRD peak intensity can signify variations in the crystallite size, crystal structure, or lattice parameters of a material. For instance, an increase in mean crystallite size is often accompanied by an increase in XRD peak intensity [52]. Additionally, high-intensity peaks in XRD patterns at specific planes indicate low interplanar distances and closely packed atoms on those planes [53]. The texture coefficient, which is calculated based on measured relative intensity in XRD patterns, provides insights into the preferred orientation of crystallites in a material [54]. Post-corrosion XRD peaks of the alloy before heat treatment (Figure 11a) are at 19.20°; post-corrosion XRD peaks of the homogenized alloy (Figure 11b) are at 18.85°; and it was observed that the post-corrosion XRD peaks of the aged alloy (Figure 11c) started at 17.34°. While the beginning of the XRD peaks of the alloys begins with MgO + Al_2_O_3_, an Mg_2_SiO_4_ peak was also observed beginning in the alloy before heat treatment. Post-corrosion XRD peaks were found to be common in the alloys SiO_2_, MgO, and MgO + Al_2_O_3_. While there is extra MgO + Al_2_O_3_ in the alloy (44.25°) before the heat treatment, Al_2_O_3_ peaks were common after homogenization (38.20°) and aging (44.66°). XRD peaks of the alloy before heat treatment were at 88.50°; XRD peaks of the homogenized alloy were at 83.15°; and it was observed that the XRD peaks of the aged alloy ended at 84.90°.

Research has shown that the presence and distribution of micro-defects play a role in the structural characteristics of the oxide film formed on aluminum surfaces, affecting the intensity of corrosion attacks [55]. The formation of an oxide layer primarily composed of Al_2_O_3_ on aluminum surfaces significantly affects the corrosion behavior of the alloys. This oxide layer can either enhance or adversely influence the pitting corrosion resistance of aluminum alloys [55]. Oxide layers have been shown to inhibit corrosion by creating stable and compact corrosion products that act as barriers against further corrosion [56]. Conversely, incomplete oxide films can decrease corrosion resistance, leading to accelerated corrosion rates even with increasing oxide film thickness [57]. The presence of MgO + Al_2_O_3_ oxide in aluminum alloys plays a crucial role in influencing corrosion behavior. The composition and characteristics of the oxide layer significantly impact the corrosion resistance of the alloys, with protective oxide layers containing Al_2_O_3_ + MgO contributing to improved performance and passivity against corrosion. Furthermore, studies have highlighted the importance of the oxide layer composition in enhancing corrosion resistance, with the formation of more protective oxide layers containing Al_2_O_3_ + MgO contributing to improved performance [58,59]. When considering the effect of Mg_2_SiO_4_ oxide on corrosion in aluminum alloys, it is essential to understand how different oxide layers interact with the environment to either enhance or inhibit corrosion. Research has shown that alloying elements, including magnesium and silicon oxides, can impact the passivity of aluminum alloys by stabilizing oxide layers [60]. These oxide layers, although thinner with increased alloying element contents, contribute to increased corrosion resistance by forming protective barriers against corrosive agents [60]. Similarly, the presence of Mg_2_Si precipitates in aluminum alloys tends to form protective oxides like SiO_2_, MgO, and SiO_2_ + MgO, contributing to enhanced corrosion resistance [61].

**Figure 12:**
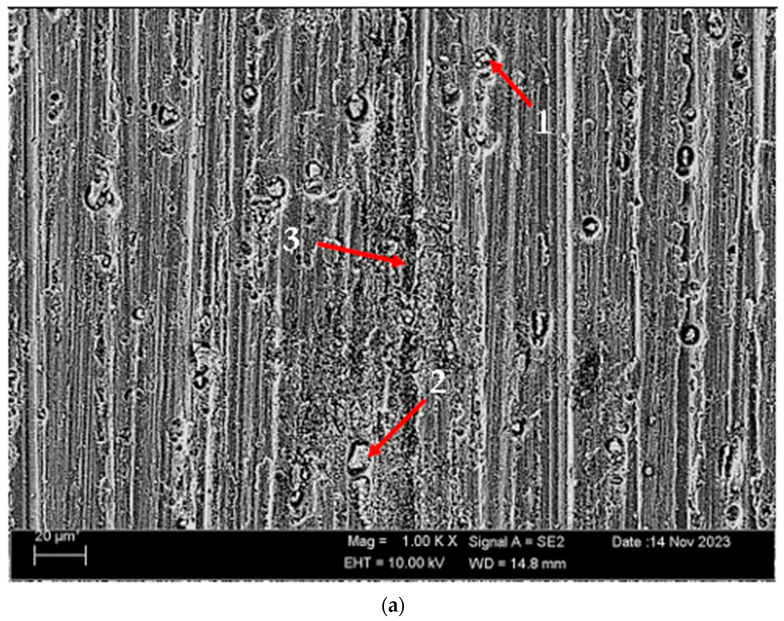

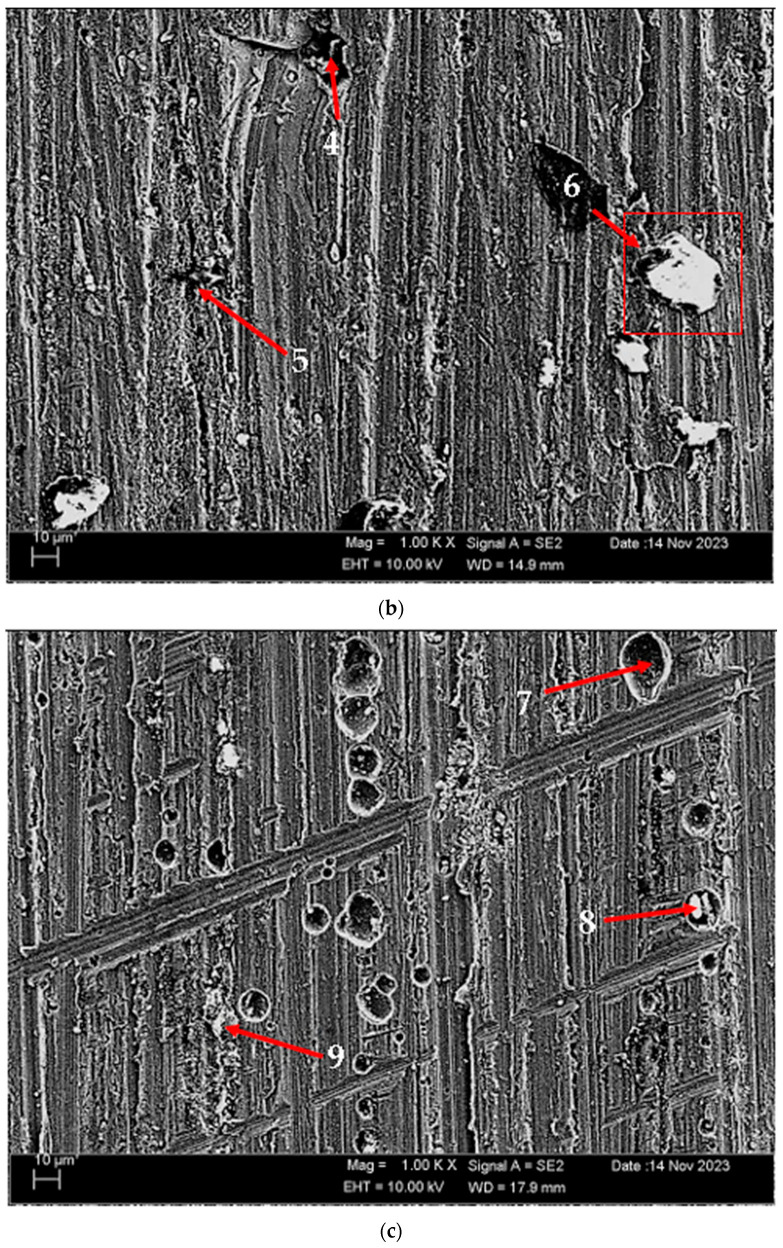
SEM micrographs of corroded H111 hot-rolled AA5754 alloys (immersion corrosion): (**a**) No heat treatment, (**b**) Homogenized, and (**c**) Aged.

Figure 12 shows the SEM micrographs of corroded (immersion corrosion) alloys before heat treatment, and after homogenization and aging. Table 4 shows the EDX analysis of the second stages with different morphologies (1–9) in Figure 12a–c. In general, when looking at the SEM micrographs (Figure 12), it is noteworthy that the pit-shaped/porous structures on the corrosion surfaces proliferate and increase in size. In the alloy that was corroded before the heat treatment (Figure 12a), polygonal gray-contrast-colored structures are seen in the pit at point 1. These structures are thought to have MgO, SiO_2_, and Al_2_O_3_ phases. At point 2, there is a situation similar to point 1, but this time, a larger rectangular-like structure in the pit attracts attention. This phase is thought to be MgO + Al_2_O_3_. At point 3, there are small pits in a sediment-like formation. Here, it is assumed that MgO + SiO_2_, Al_2_O_3_, MgO + Al_2_O_3_, and Mg_2_SiO_4_ phases are present. It is thought that the peak-like structures remaining in the deep pit at point 4 in the corroded alloy after homogenization (Figure 12b) are Al_2_O_3_ and MgO. It is assumed that the phases thought to be present at point 5 at point 4 are also seen here. At this point, pits were seen in a dense arrangement on a foliation-like surface. Point 6 is the richest point in oxygen and it is thought that there are MgO + Al_2_O_3_, Mg_2_SiO_4_, and MgO + SiO_2_ phases here. The small cylinder-like structures in the deep crater at point 7 in the corroded alloy after aging (Figure 12c) are thought to be MgO. It is assumed that the rectangular/cylindrical structures covering the pit at point 8 are Al_2_O_3_ and SiO_2_. It is thought that the leaf-shaped structures at point 9 are more pointed and the deposits around it may consist of Al_2_O_3_, SiO_2_, and MgO.

#### 3.5.2. Potentiodynamic Polarization (PD) Tests

**Figure 13:**
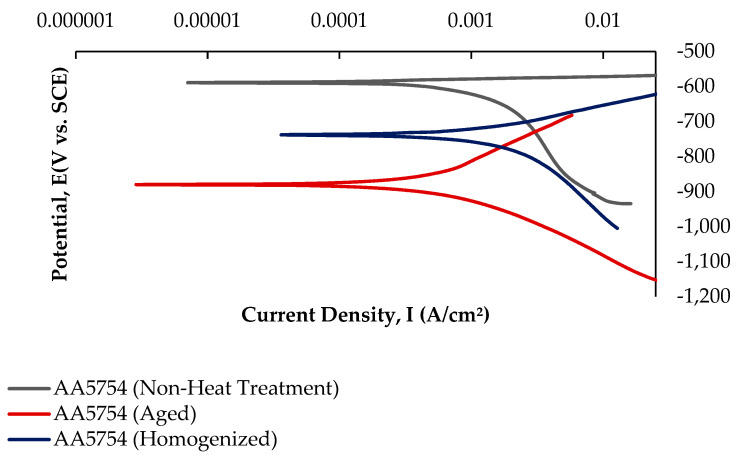
Potentiodynamic current–potential curves of H111 hot-rolled AA5754 alloy before heat treatment, and after homogenization and aging.

Potentiodynamic polarization curves are crucial for determining parameters such as corrosion potential, corrosion current density, and corrosion rate, making them a rapid and effective method for evaluating corrosion behavior [62]. While Figure 13 shows the current–voltage curves of the H111 hot-rolled AA5754 alloy before heat treatment, and after homogenization and aging, Table 5 lists its corrosion data. As seen in Figure 13 and Table 5, there is a strong decrease in the corrosion current densities (Icorr) of the alloy before heat treatment compared with other alloys. The alloy that was corroded before heat treatment showed the best corrosion behavior by creating a corrosion potential of 1.04 ± 1.5 V at a current density of −586 ± 0.04 μA/cm^2^. However, after aging, the corroded alloy showed the worst corrosion behavior with a corrosion potential of 5.16 ± 3.3 V at a current density of −880 ± 0.01 μA/cm^2^.

The intermetallic phases Al_3_Mg_2_ and Al_6_Mn play a significant role in the mechanical properties and corrosion behavior of aluminum alloys. Li [63] found that Al_3_Mg_2_ exhibits the active dissolution of both Al and Mg elements at low pH, while selective dissolution occurs at higher pH, affecting the corrosion resistance of AA5000 series alloys. Similarly, Jin [64] observed that the addition of Mg to Al-Si coatings promoted the formation of Al_3_Mg_2_, which reduced corrosion resistance. Yao [65] found that the addition of Mn to Mg-3Al alloys improved corrosion resistance by encapsulating detrimental phases. Lachowicz and Jasionowski [66] observed a significant influence of the Mg_2_Si intermetallic phase on the progression of corrosion. The occurrence of localized corrosion on the surface of a material, specifically around well-formed Chinese script-like precipitates, indicates either the anodic behavior of the Mg_2_Si phase or a deterioration of the protective surface layer [67]. Yasakau et al. [68] reported that β-(Al_3_Mg_2_) phases, together with Mg_2_Si precipitate, exhibit anodic activity. They also observed that these phases dissolve during corrosion, and the corrosion process is further accelerated by the deterioration of the protective oxide film.

**Figure 14:**
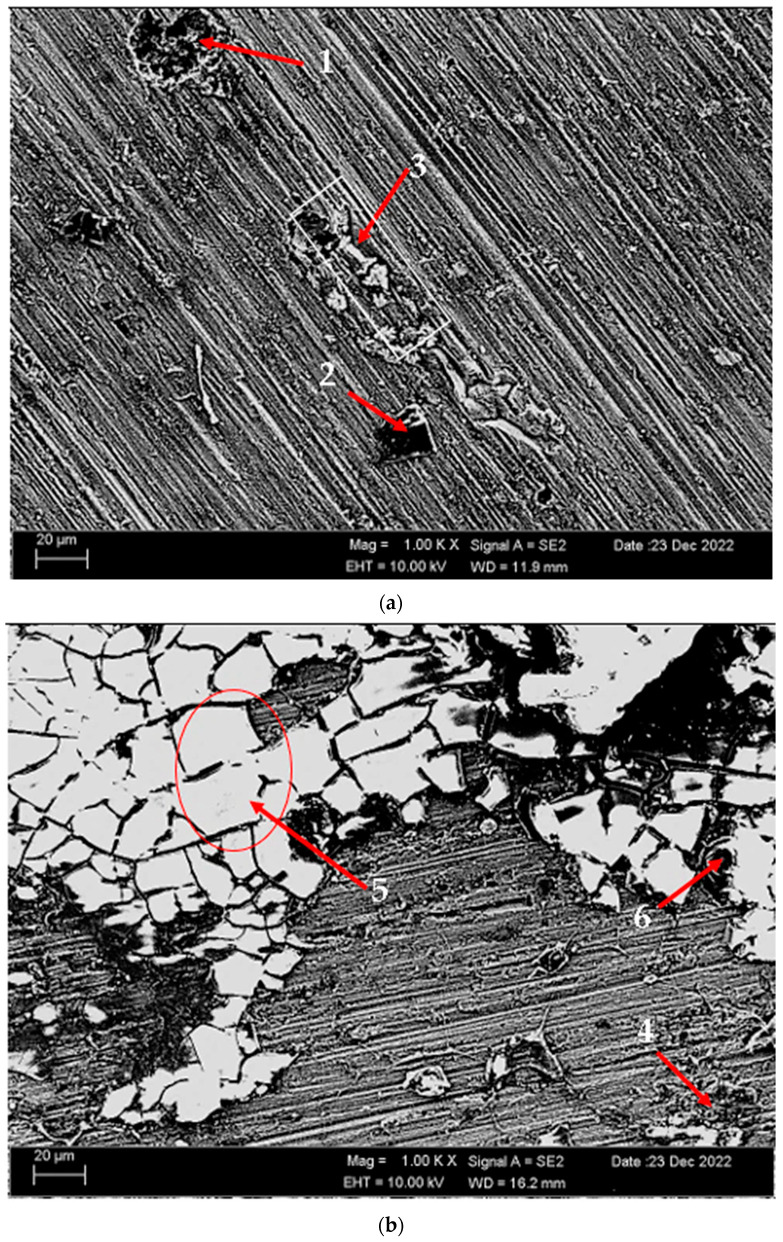

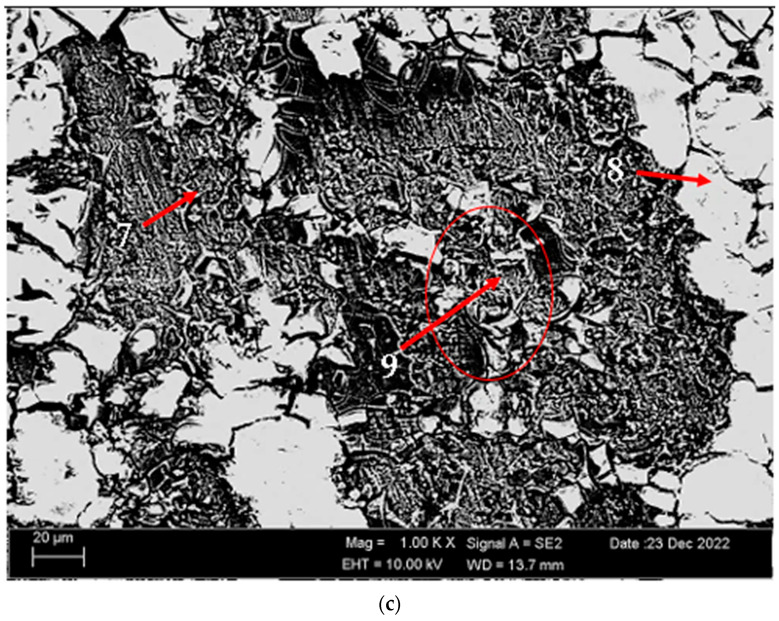
SEM micrographs of corroded H111 hot-rolled AA5754 alloys (potentiodynamic polarization cor-rosion): (**a**) No heat treatment, (**b**) Homogenized, and (**c**) Aged.

Figure 14 shows SEM micrographs of corroded (potentiodynamic polarization) alloys before heat treatment and after homogenization and aging. Table 6 shows the EDX analysis of the second stages with different morphologies (1–9) in Figure 14a–c. In general, when looking at the SEM micrographs, it was observed that the alloys corroded with the formation of more porous areas and cracked oxides after homogenization and aging. The potentiodynamic polarization test and immersion test support each other (See Figure 10). In the alloy that was corroded before the heat treatment (Figure 14a), triangular-shaped structures formed within the pitting can be seen at the first point. These structures are thought to have MgO, SiO_2_, Mg_2_SiO_4_, and Al_2_O_3_ phases. At the second point, there are small round-shaped light-gray contrast-colored structures on the rectangular-shaped structure. The phases here are considered as MgO, SiO_2_, and Al_2_O_3_. It is thought that the spiny structures at point 3 are MgO + SiO_2_, Al_2_O_3_, and MgO. It is thought that the obvious band-shaped precipitation at the fourth point in the corroded alloy after homogenization (Figure 14b) is MgO, SiO_2_, and Mg_2_SiO_4_. The large structures with light-gray contrast at point 5 are thought to be MgO + SiO_2_, Al_2_O_3_, and Mg_2_SiO_4_. The crater structure formed at point 6 is thought to be MgO and Al_2_O_3_. It is thought that the formation of MgO and Al_2_O_3_ film with branched cracks (Figure 14c) at the seventh point of the corroded alloy after aging increased corrosion. The light-gray contrast MgO, SiO_2_, and Al_2_O_3_ at point 8 and the crater-like MgO + SiO_2_, Al_2_O_3_, and MgO oxides at point 9 containing branched cracks also increased the corrosion rate. The presence of a thicker oxide covering effectively prevented corrosion, whereas the thinner oxide film vanished and its underlying material experienced corrosion. This corresponds to the breaking of the oxide film and the shedding of second-phase practices on the interfaces [57]. In Figure 14c, the distributed pitting formation on the surface of the aged H111 hot-rolled AA5754 alloy after the immersion corrosion test explains the high corrosion rate in the aging heat-treatment condition.

After the corrosion test of the unheat-treated H111 hot-rolled AA5754 alloy, it was observed that MgO, SiO_2_, Mg_2_SiO_4_, and Al_2_O_3_ oxide films were formed on the surface. The formation of oxide films on Al-Mg alloys, including the AA5754 alloy, is a complex process influenced by factors such as heat treatment, humidity, and the presence of other elements. Lea [69] and Yoon [70] both discuss the formation of MgO and Al_2_O_3_ oxide films on the surface of Al-Mg alloys, with Yoon [70] specifically noting the formation of MgAl_2_O_4_ spinel as a secondary oxide. Lea [69] found that the oxidation of Al-Mg alloys during heat treatment results in the formation of a thin self-healing amorphous film of A1_2_O_3_, which then transforms into a magnesium-rich surface with an island MgO film. Fernández [71] further explores the influence of films and the presence of other elements on the oxidation and corrosion of these alloys, offering vital insights into the underlying mechanisms. These studies collectively suggest that the formation of MgO, SiO_2_, Mg_2_SiO_4_, and Al_2_O_3_ oxide films on the corroded surface of the unheat-treated H111 hot-rolled AA5754 alloy is a result of the complex oxidation process influenced by the alloy composition and environmental conditions. Aluminum alloy, a lightweight metal, is used for real-world applications such as the automotive and aerospace industries, and has many advantages such as good corrosion resistance, good economic benefits in product life, and a high recycling utilization rate [72]. The use of aluminum alloy fuel tanks for the automotive industry not only reduces the consumption of limited resources but also benefits environmental protection. The application of aluminum alloy parts is in green production, which fits the current international trends in automobile manufacturing [73]. AA 5754-H22 alloy has become widely used in the ship building and aircraft industries due to its tensile properties, bending behavior, and hardness. It is used due to its high corrosion resistance and its good weldability. AA 5754 alloy is also preferred because of its resistance to sea water and chemicals [74]. Due to the fact that they have a good combination of strength and formability, in addition to having great corrosion resistance, AA 5xxx series alloys are utilized in automotive applications for the purpose of constructing sections of car physiques and chassis [75,76].

## 4. Conclusions

The following results are given regarding the microstructural properties, wear resistance, and corrosion behavior of H111 hot-rolled AA5754 alloy before heat treatment and after homogenization and aging:

- The as-rolled material exhibits a dense pancake-shaped grain structure, which is typical of as-rolled material. Observation along the L-direction did not yield distinct demarcations among grains and there was no uniform distribution, with precipitates at the grain boundary. When they are aged, there is a parallel increase in fine and huge black and gray contrast particles in the zone. Therefore, it could be stated that the amount of fine grains increased due to the rise in the homogenization process. The rolled base metal with the grain orientation was found to be parallel to the rolling direction. On the other hand, the coarse grains were clearly observed in the aging heat-treatment condition. The grains have an elongated morphology consistent with the rolling process of the metal before the heat-treatment process.
- The aged alloy has the highest hardness with a value of 86.83 HB; the lowest hardness was seen in the alloy before heat treatment with a value of 68.67 HB.
- The weight loss and wear rate of this material at the end of 10,000 m are, respectively, 1.01 × 10^−3^ g and 5.07 × 10^−9^ g/Nm. It was observed that the alloy had the highest weight loss and worst wear resistance before heat treatment. Weight loss and wear rates at the end of 10,000 m are, respectively, 3.42 × 10^−3^ g and 17.08 × 10^−9^ g/Nm. According to these results, the friction coefficients during wear are parallel and the material with the lowest friction coefficient after aging is 0.045.
- While the alloys corroded after aging showed more weight loss, the alloys corroded before heat treatment exhibited better corrosion behavior. Among the alloys, the least weight loss after 24 h was observed in the alloy that was corroded before heat treatment and this value was 0.69 × 10^−3^ mg/dm^2^. The highest weight loss was observed in the aged alloy with a value of 1.37 × 10^−3^ mg/dm^2^. The alloy before heat treatment, which corroded after casting, showed the lowest corrosion rate with a value of 0.39 × 10^−3^ mg/(dm^2^·day) after 72 h.
- The alloy that was corroded before heat treatment showed the best corrosion behavior by creating a corrosion potential of 1.04 ± 1.5 V at a current density of −586 ± 0.04 μA/cm^2^. However, after aging, the corroded alloy showed the worst corrosion behavior with a corrosion potential of 5.16 ± 3.3 V at a current density of −880 ± 0.01 μA/cm^2^.

## Figures and Tables

**Table 1:** Compositions of alloys (in wt%).

Alloy	Chemical Composition (wt.%)					
	Mg	Mn	Si	Fe	Cr	Al
AA5754	3.650	0.526	0.349	0.348	0.122	Bal.

**Table 2:** Figure 3 (1–9) EDX phases (wt%).

Points	Mg	Al	Si	Cr	Mn	Fe
1	4.50	93.00	-	-	1.21	1.29
2	5.05	94.30	-	0.15	-	0.50
3	4.88	93.78	0.44	0.15	-	0.63
4	1.21	69.43	4.60	0.46	7.22	17.08
5	4.76	93.53	1.29	0.09	-	0.32
6	4.86	94.44	-	-	0.71	-
7	2.06	33.29	63.76	0.28	0.61	-
8	4.96	94.34	0.02	0.06	0.35	0.28
9	4.22	91.49	0.51	-	1.37	2.42

**Table 3:** EDX findings of Figure 8(1–9) (wt.%).

Points	C	O	Mg	Al	Si	Mn	Fe
1	3.685	23.795	3.815	66.53	0.11	0.28	1.785
2	5.21	37.57	3.11	53.94	0.17	-	-
3	5.04	6.08	4.74	82.35	0.24	0.69	0.85
4	2.58	26.945	3.61	66.02	0.145	-	0.705
5	2.82	28.58	3.365	64.99	0.04	0.21	-
6	3.77	12.41	4.01	78.80	-	0.54	0.46
7	7.32	26.845	3.89	61.085	0.695	-	0.165
8	6.315	38.785	3.55	50.80	0.495	0.05	0.05
9	9.12	25.73	3.99	60.60	0.54	-	0.03

**Table 4:** EDS findings of Figure 12(1–9) (wt.%).

Points	O	Na	Mg	Al	Si	Cl	Mn	Fe
1	6.433	0.603	2.226	75.33	2.403	0.183	4.326	8.50
2	5.63	1.20	2.345	75.855	2.445	0.645	4.38	7.495
3	19.98	1.32	2.91	71.185	1.265	1.13	0.60	1.605
4	6.936	0.53	4.486	87.253	0.29	0.343	0.163	-
5	14.42	2.255	3.705	76.245	0.87	2.505	-	-
6	49.933	12.263	2.113	16.863	1.96	13.13	1.186	2.556
7	5.458	0.37	4.30	87.666	1.014	0.09	0.534	0.564
8	40.52	0.66	2.09	47.60	1.45	0.81	3.00	3.87
9	19.67	0.625	7.235	70.54	1.10	0.665	0.165	-

**Table 5:** Electrochemical corrosion data results of indicated in Figure 13.

Alloys	Ecorr (V)	Icorr (μA/cm^2^)
AA5754 (Non-Heat Treatment)	−586 ± 0.04	1.04 ± 1.5
AA5754 (Homogenized)	−736 ± 0.01	2.04 ± 2.0
AA5754 (Aged)	−880 ± 0.01	5.16 ± 3.3

**Table 6:** EDS findings of Figure 14 (1–9) (wt.%).

Points	O	Na	Mg	Al	Si	Cl	Mn	Fe
1	35.43	3.25	8.65	43.32	8.02	0.84	0.50	-
2	43.33	9.24	4.43	30.71	2.42	5.53	1.39	2.96
3	24.18	1.70	2.49	52.48	4.09	0.40	5.36	9.30
4	40.85	0.63	3.18	38.82	14.85	1.19	0.37	0.12
5	62.15	1.70	4.47	21.63	7.99	0.89	1.18	-
6	44.87	0.70	4.40	49.11	0.07	0.45	0.08	0.32
7	14.76	0.56	4.18	80.15	-	0.01	-	0.35
8	59.13	0.71	3.72	26.92	0.26	7.74	0.51	1.00
9	39.77	0.51	3.80	52.87	0.48	1.96	0.48	0.12

## Data Availability

No new data were created or analyzed in this study. Data sharing is not applicable to this article.
